# Congenital Diaphragmatic Hernia: Review of Current Concept in Surgical Management

**DOI:** 10.5402/2011/974041

**Published:** 2011-12-20

**Authors:** Emeka B. Kesieme, Chinenye N. Kesieme

**Affiliations:** ^1^Department of Surgery, Irrua Specialist Teaching Hospital, PMB 8, Edo State, Irrua, Nigeria; ^2^Department of Paediatrics, Irrua Specialist Teaching Hospital, PMB 8, Edo State, Irrua, Nigeria

## Abstract

Congenital diaphragmatic hernias (CDHs) occur mainly in two locations: the foramen of Morgagni and the more common type involving the foramen of Bochdalek. Hiatal hernia and paraesophageal hernia have also been described as other forms of CDH. Pulmonary hypertension and pulmonary hypoplasia have been recognized as the two most important factors in the pathophysiology of congenital diaphragmatic hernia. Advances in surgical management include delayed surgical approach that enables preoperative stabilization, introduction of fetal intervention due to improved prenatal diagnosis, the introduction of minimal invasive surgery, in addition to the standard open repair, and the use of improved prosthetic devices for closure.

## 1. Introduction

The estimated incidence of congenital diaphragmatic hernia is 1 in 2000–5000 live births [1].

The aetiology of congenital diaphragmatic hernia is unknown, however, 2% of cases have been noted to be familial and another 15% of patients have associated chromosomal abnormalities [2].

Pulmonary hypoplasia is an important defect in congenital diaphragmatic hernia [2], and the severity of this pathology is largely dependent on the degree of pulmonary hypoplasia, pulmonary hypertension, and associated malformations.

There have been recent advances in the medical management and postnatal care, all targeting the primary pathophysiological mechanism, and in the surgical management with the introduction of fetal interventions, laparoscopic repair, and lung transplantation. The treatment focus has also changed from emergency surgery at birth or presentation to surgical closure of defect after stabilization of the patient.

Despite all these advances in neonatal care and surgical management, congenital diaphragmatic hernia (CDH) remains a condition with a significantly high mortality rate [3].

We aim to update clinicians in the recent advances in the diagnosis and surgical management of patients who present with Bochdalek hernia and Morgagni hernia.

## 2. Methods

A literature search on the subject was done from 1970 till date using manual library search and journal publications on Pubmed/Medline, Google scholar, and EMBASE.

We used the following keywords: congenital diaphragmatic hernia, Bochdalek hernia, Morgagni hernia, retrosternal, and posterolateral hernia. Full texts of the materials, including those of relevant references were collected and studied. Information relating to the epidemiology, prenatal diagnosis and fetal interventions, postnatal diagnosis, investigations and surgical treatment (open, laparoscopic, and robotic) was extracted from the materials. The most relevant reported case series, case reports, and literature review were used for this study.

## 3. Results

### 3.1. Pathoanatomy/Epidemiology

Bochdalek popularized the concept of herniation due to failed closure of the primitive communication between the pleura and the peritoneal cavity (pleuroperitoneal duct), while Morgagni described a hernia that occur through a defect resulting from failure of the anterior pleuroperitoneal membrane to fuse with the sternum and costal cartilages during embryogenesis [4, 5]. Anatomically, herniation through the right sternocostal triangle is actually the Morgagni hernia while the one through the left sternocostal triangle is Larry Hernia [6].

These herniations were initially thought to be responsible for pulmonary hypoplasia by compression of the lungs, more on the ipsilateral side. Lung hypoplasia may be unrelated or partially related to the diaphragmatic defect. Nitrofen has been shown to interfere with early lung development before and separate from development of the diaphragm. Keijzer et al. postulated the dual-hit hypothesis, which explains pulmonary hypoplasia in CDH by two insults, one affecting both lungs before diaphragm development and one affecting the ipsilateral lung after defective diaphragm development [7].

Morgagni hernia usually presents in the paediatric population. It is rarely diagnosed in adulthood. Three percent (3%) of congenital diaphragmatic hernias are the Morgagni type, and only 4% present bilaterally [8]. Bochdalek hernia is occasionally bilateral, and it occurs on the left side in 80% of cases [9]. This is because the right pleuroperitoneal canal closes earlier and the liver buttresses the right hemidiaphragm. In a study on incidentally discovered Bochdalek hernia in an adult population, Mullins et al. reported that sixty-eight percent (68%) of hernia occur on the right side, 18% occur on the left side, and 14% occur bilaterally [10]. The contents of these hernias may include fat, omentum, or solid or hollow viscera (spleen, small intestine, or large intestine). A female predominance for Bochdalek hernia observed by some authors has been well documented [10, 11], however others have observed a male predominance [12, 13].

### 3.2. Prenatal Diagnosis and Fetal Intervention

Diagnosis of congenital diaphragmatic hernia can be made prenatally or after birth. In developed countries, a high prenatal detection rate of congenital diaphragmatic hernia (59%) has been reported and the gestational age at diagnosis was greater than 24 weeks in half of the prenatally diagnosed cases [14]. However, prenatal detection of CDH is rare in developing countries due to inadequate facilities [15]. Ultrasonographic features in keeping with this condition include maternal polyhydramnios, an absent or intrathoracic stomach bubble, a mediastinal and cardiac shift away from the side of the herniation, a small fetal abdominal circumference, and rarely fetal hydrops [16]. Patients diagnosed prenatally will need to undergo additional imaging studies to evaluate some prognostic factors and genetic studies to rule out associated congenital anomalies. Prenatal diagnosis is not possible in those whom herniation of abdominal viscera into the chest took place presumably just at delivery through a small diaphragmatic defect [16]. Useful prognostic tool that can be measured include the fetal lung area to head circumference ratio, the position of the liver and stomach using 3D-ultrasound scan, and measurement of fetal lung volume using magnetic resonance imaging. Jani et al. revealed that a low lung area to head circumference ratio (less than 1) and herniation of the liver are predictive of poor survival [17]. Fetal surgery has been explored in experimental studies and has been applied clinically. In experimental studies, prenatal tracheal occlusion has been shown to induce lung growth with reduction of herniated viscera and a dramatic improvement in lung compliance and gas exchange [18]. Fetal surgery has revolutionized from open surgical repair to tracheal occlusion techniques: open surgical tracheal occlusion (TO), endoscopic external tracheal occlusion, and endoscopic endoluminal tracheal occlusion [15]. Open fetal surgery to correct the diaphragmatic defect has been abandoned. A substantial improvement in outcome and survival of patients has been reported with fetal endoscopic tracheal occlusion, though it is still associated with a high incidence of preterm prelabor rupture of membranes and preterm delivery in severe congenital diaphragmatic hernia [19]. The main advantage of prenatal detection of congenital diaphragmatic hernia may be the facilitation of postnatal care as the mother can be referred to a specialist centre where they can be electively managed by fully prepared team of neonatologists and surgeons [20].

### 3.3. Postnatal Diagnosis

Postnatal presentation of Bochdalek hernia may be symptomatic or asymptomatic and may be discovered as an incidental finding. However, they may be diagnosed later in adulthood with nonspecific respiratory and gastrointestinal symptoms and signs. Acute presentation of congenital diaphragmatic hernia include severe immediate cardiorespiratory distress with cyanosis, tachypnea, tachycardia with findings of a prominent hemithorax with minimal air entry, a displaced apex beat indicating mediastinal shift, and often a scaphoid abdomen [21].

For those that present later in life, the most frequent features include respiratory symptoms (43%), gastrointestinal symptoms (33%), both respiratory and gastrointestinal symptoms (13%), and asymptomatic (11%) [22]. The majority of right-sided lesions present with respiratory symptoms, while for left sided lesions, equal incidence of respiratory and GI symptoms have been observed [21, 23]. Clinical symptoms may include upper abdominal pain, bloating, discomfort after meal, vomiting, cough, dyspnea, and palpitation. Bochdalek hernia may present late or may present with complications such as gastric volvulus, splenic rupture, gastric or other intestinal obstruction, and/or perforations [24–27].

### 3.4. Investigations

**Figure 1:**
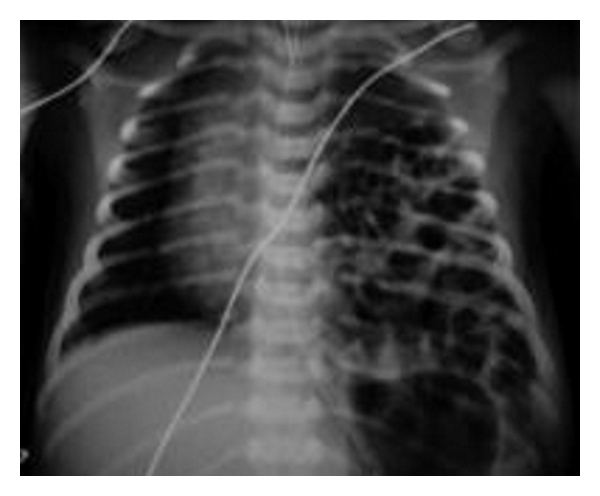
Chest radiograph showing intestinal loops in the left thoracic cavity.

**Figure 2:**
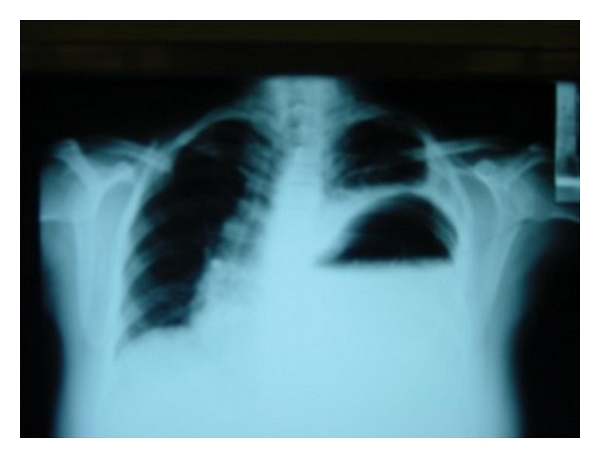
Chest radiograph showing the stomach in the left thoracic cavity.

Radiological investigations are usually required to confirm diagnosis, assess the contents of the hernia, and evaluate the presence of any associated abnormality. Chest radiograph may reveal an anteromedial mass in Morgagni hernia. Other findings include the presence of stomach or loops of bowel in the abdomen (Figures 1 and 2) or solid paracardiac shadow if omentum is herniating and the presence of mediastinal shift. A change in the intrathoracic gas pattern may be revealed by repeated chest radiographs. The presence of gas or air fluid level in the chest has led to erroneous diagnosis of pneumothorax or pleural effusion in 25% of cases [23], prompting inappropriate thoracentesis or chest tube insertion, and subsequent inadvertent perforation of the herniated viscera [21]. Inserting a radioopaque nasogastric tube will clearly delineate the presence of stomach in the chest. The position of herniation and contents is best delineated by the computerized tomographic scan which can also help to rule out differential diagnosis such as mediastinal masses, bronchogenic cyst, and the other congenital malformations of lung parenchyma [21, 28]. Temizöz et al. revealed that the multiplanar and reconstruction features of multidetector CT facilitated the diagnosis of asymptomatic incidental Bochdalek hernia in their series [13]. 

In elective cases, contrast gastrointestinal studies (barium meal or enema) can be performed to confirm diagnosis. In patients with associated malrotation due to Ladd's band who are not acutely ill, malrotation can be diagnosed preoperatively by barium meal (to identify duodenal position) or barium enema (to identify caecal position) [29]. This is important because it will largely determine the surgical approach; in which case, transabdominal route is recommended. Chen et al. demonstrated the capability of magnetic resonance imaging (MRI) in visualizing diaphragmatic discontinuity and in showing connection between bowel segments in the abdomen and chest of an 11-month-old baby with delay onset of congenital diaphragmatic hernia (CDH) [30]. Other investigations that can be relevant in patients with CDH include cardiac, renal, and cranial sonography.

### 3.5. Surgical Treatment

Emergency surgical repair of congenital diaphragmatic hernia was advocated before the 1980s. No improvement in gas exchange was observed and thoracic compliance and PaC0_2_ had a tendency to deteriorate in the immediate postoperative period [31]. Hence the concept has changed from performing emergency repair to delaying repair for at least 24–48 hours to allow for clinical stabilization and a fall in pulmonary vascular resistance [2, 32]. Depending on the clinical condition of the patient, surgery can be delayed for up to 7–10 days.

The timing of surgery should be dependent on when the patient's clinical condition is optimized rather than adhering to a specific time period. Hence the mean age of repair remains variable [33]. Though some studies have revealed no clear evidence which favours delayed (after stabilization) as compared to immediate repair (within 24 hours) [34], others have proven that early repair in the face of labile respiratory and unstable haemodynamic function is harmful, and delayed operation may allow patients with borderline prognosis to survive [35]. There is no evidence that timing of surgery influence survival, however, associated conditions (cardiac defects and renal failure) and initial blood gases are significant factors that influence survival [36].

Generally, repair of congenital diaphragmatic hernia can be performed safely and effectively using different approaches [37]. Some authors advocate transthoracic approach [38] or transabdominal approach [39], while others advocate video-assisted thoracoscopic [40] or laparoscopic techniques [5, 41]. Transthoracic approach provides wide exposure and easy repair of hernia sac in Morgagni hernia [38]. It provides a better access to the hernia sac in obese patients, and it is preferred for right-sided hernia because it allows better visualisation of the diaphragmatic foramen and adhesions around the pleura and pericardium [37]. Transabdominal approach makes it easy to reduce the hernia content and repair the sac [39]. It is also regarded as being technically better for repairing bilateral and complicated hernias [37]. Transabdominal approach is mandatory in complications (strangulation, incarceration, or perforation with peritonitis) as it is strongly recommended that the entire abdominal cavity be inspected in all cases of peritonitis [37, 39]. Laparascopic repair has been described as being safe and reliable and an excellent way to confirm diagnosis and repair noncomplicated hernia of Morgagni [42]. It also has all the advantage of minimal invasive surgery including shortened postoperative stay, reduction in trauma, faster return to normal activity, and diet and minimal or no postoperative complication [5]. Thoracoscopic approach is also a minimal access route and has an additional advantage of affording the surgeon more choices in the selection of an extrathoracic ligation method and allowing the surgeon to make a proper incision and precise repair of the defect [43]. In management of adult patients with Bochdalek or Morgagni hernia, Nakashima et al. recommended thoracoscopic approach following successful video-assisted repair in patients with severe adhesions of the hernia sac to the parietal pleura and diaphragm [43]. Mousa et al. also reported a hand-assisted intracorporeal thoracoscopic repair of congenital diaphragmatic hernia [44]. Shah et al. advocated thoracoscopic approach for neonatal Bochdalek, and Marhuenda et al. advocated laparoscopic approach for Morgagni hernia [45, 46]. Delayed presentation and a symptom free interval with negligible respiratory symptoms are the indications for laparoscopic repair in children with Morgagni hernia [47]. Morgagni hernia should not be repaired through the laparoscopic route in neonates with respiratory distress due to the risk associated with CO_2_ pneumoperitoneum [48].

Other approaches that have been described in the literature include transsternal approach, which has been advocated in patients with concomitant congenital cardiac anomaly undergoing repair under cardiopulmonary bypass or hernia repair in patients undergoing coronary artery bypass surgery [49]. A transxiphoid approach after thoracoscopic view can be used to dissect adhesions or to repair the defect [46, 50].

Excision of hernia sac in Morgagni hernia is a debatable issue and hence may largely depend on the skill of the surgeon, the presentation of the individual patient per se and if surgery can be technically safely guaranteed as in excision of small sac with no intrathoracic adhesion [51]. Some authors advocate against excision of hernia sac because of risk of massive pneumomediastinum, damage to mediastinal structures, and cardiorespiratory complications [52, 53]. However, Gadacz et al. encouraged sac excision on account of the advantage of reduction of tissue trauma because only the sac is manipulated (rather than its contents) in cases where the colon or stomach are contained within the sac and the possibility for transmural visceral injury or neurovascular injury exists, decreased chance for symptomatic fluid collection since the serous lining membrane is removed and sac excision also negates the chance that the sac itself can act as a lead point for recurrent herniation [54].

**Figure 3:**
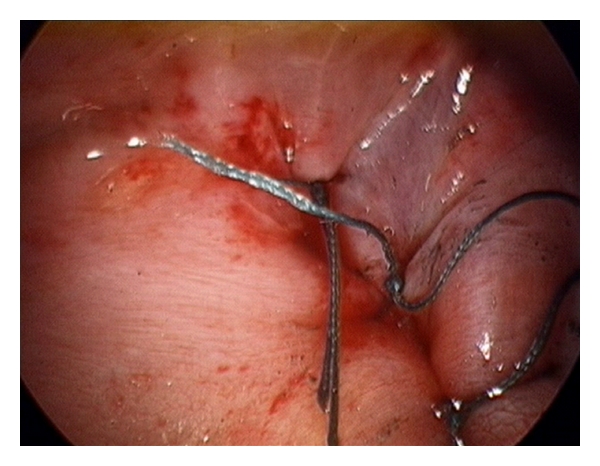
Primary repair.

Herniorrhaphy is either carried out primarily or in cases in which most of the hemidiaphragm is lacking, using a piece of prosthetic mesh or muscle flap. Primary repair is performed when there is sufficient diaphragm to approximate without tension (Figure 3). The advantages of primary repair in neonates include low recurrence rate and avoidance of mechanical and infectious complications associated with implanted prostheses [55]. Patch closure with either prosthetic mesh or autologous/biological graft is necessary to achieve tension-free repair in large congenital diaphragmatic hernia. The need for a patch repair has been shown to be an independent predictor of mortality and was independently associated with secondary outcome measures of morbidity including the need for oxygen at discharge and the duration of ventilation [56]. Those requiring patch repair had a significant higher morbidity [56]. Generally, the use of prosthetic material is complicated by granulation, allergic reaction, infection, recurrence of hernia, and thoracic deformity [57].

Prosthetic materials, including polypropylene mesh (Marlex) [58], polytetrafluoroethylene (PTFE) patch (Goretex) [59], expanded polytetrafluoroethylene (ePTFE) [60], and polyethylene terephthalate mesh (Dacron) [61] have been used for repair of large congenital diaphragmatic hernias. Others that have reportedly used Surgisis [62], dura [63], bovine pericardium [64], autologous tissues such as fascia [65] or muscle flaps [66–68].

Prosthetic materials demonstrate a wide variety of characteristics with individual benefits and demerits. However, none has been described as an ideal prosthesis. Most of the existing studies comparing the characteristics of these prostheses have largely been animal studies.

Novitsky et al. compared adhesion formation, tissue ingrowth, and textile characteristics one year after intra-abdominal placement of polypropylene, ePTFE, ePTFE/PP, and reduced weight PP and oxidized regenerated cellulose (rPP/C) [69]. ePTFE was found not to induce adhesion. It was also most compliant and shrank more than the other types of mesh [69, 70]. Polypropylene caused most adhesion than others [69, 70]. Formation of adhesion may be critically related to the pore size as the macroporous polypropylene mesh promoted adhesion formation unlike microporous nature of the visceral side of ePTFE [71]. Gonzalez et al. compared absorbable (small intestinal mucosa) and nonabsorbable mesh repair in the repair of large congenital diaphragmatic hernia. Absorbable mesh allows better integration to the chest wall, more muscle growth into the newly formed tissue providing a more natural and durable repair and less fibrotic reaction than ePTFE [72]. Even among the absorbable small intestinal submucosa (SIS), 8-ply SIS has been found to be superior to 4-ply SIS [73].

In patients who had prosthetic patch repair, patch separation can occur necessitating reoperation. Reverse latissimus dorsi muscle flap with end to side neural coaptation of thoracodorsal nerve with phrenic nerve has been used for recurrences after synthetic patch closure [66, 67]. This provides an alternative to repeat prosthetic patch repair and offers the advantage of using an autologous vascularised tissue with an additional advantage of physiologic neodiaphragmatic motion due to phrenic nerve innervation [67]. Use of this flap has been feared in patients undergoing ECMO because of risk of anticoagulation and bleeding. However Brant-Zawadzki et al. have shown in their study that split abdominal wall muscle flap for herniorrhaphy is possible for patients still on ECMO [68]. Anterior abdominal wall muscle flaps provide similar short-term and long-term outcomes as prosthetic patch repair.

The drawback of the use of local muscle flap is the associated significant body wall deformity [59] and, hence, is largely restricted to recurrent CDH. However, the risk of infection is reduced, though with further risk of recurrence as a result of atrophy from denervated muscles [67].

Okazaki et al. was the first to use Toldt's fascia flap, a flap consisting of the small medial muscle remnant of the diaphragm, Toldt's fascia, peritoneum, and retroperitoneal connective tissue to repair large diaphragmatic hernia [65]. Koot et al. described the use of lyophylized dura patch in the repair of diaphragmatic defects, but they concluded that this material was not totally reliable, as they documented almost 20% recurrence of the hernia [74]. They found no recurrence among those who underwent direct closure. Bovine pericardium patch has been hypothesized to be a lasting alternative to reconstructing agenesis of the left hemidiaphragm because of the strength, elasticity, resistance to sutures and the possibility of growth [64]. Animal studies have also shown that this patch showed better tissue incorporation, lower degree of adhesions, and no graft wrinkling was observed when compared to Polypropylene mesh [75].

Patients who underwent patch repair have been noted to be significantly more likely to develop increased risk of recurrence and small bowel obstruction than patients who had a primary repair [76]. Jancelewicz et al. reported that patch repair is independently predictive not only of recurrence but also of early chest deformities. They found that the strongest predictor of small bowel obstruction was patch repair. A higher incidence of small bowel obstruction was noted in repair using absorbable patch [77]. However, Nasr et al. while comparing results of anterior abdominal wall muscle flap and prosthetic patch repair noted no difference in the short- and long-term outcomes between the two [78].

Robotic repair of diaphragmatic hernia has been described as being safe [79], although the long-term outcomes of these repairs are yet to be evaluated. Both Morgagni and Bochdalek hernia have all been successfully repaired via the use of robotic instruments [80, 81]. In a reported case of a successful robotic repair of Bochdalek hernia using the abdominal route, the articulating instruments were noted to offer more freedom in accessing the difficult posterolateral region unlike the rigid laparoscopic instruments [81].

Van Meurs et al. performed lung transplantation on a patient with congenital diaphragmatic hernia who continued to deteriorate after delayed repair despite apparent resolution of pulmonary hypertension [82]. No other report of lung transplantation for the treatment of congenital diaphragmatic hernia in the literature followed this paper.

### 3.6. Perioperative Care

Advances in perioperative care based on correcting a disorder in physiology rather than anatomy has been shown to improve survival. These include gentle ventilation (concept based on minimizing lung injury and ignoring right to left shunt), high frequency ventilation (preserves end expiratory volume and avoids alveolar overdistension), and treatment of pulmonary hypertension with inhaled nitric oxide therapy and extracorporeal membrane oxygenation (ECMO) [1].
